# Dapagliflozin and Diuretic Use in Patients With Heart Failure and Reduced Ejection Fraction in DAPA-HF

**DOI:** 10.1161/CIRCULATIONAHA.120.047077

**Published:** 2020-07-16

**Authors:** Alice M. Jackson, Pooja Dewan, Inder S. Anand, Jan Bělohlávek, Olof Bengtsson, Rudolf A. de Boer, Michael Böhm, David W. Boulton, Vijay K. Chopra, David L. DeMets, Kieran F. Docherty, Andrej Dukát, Peter J. Greasley, Jonathan G. Howlett, Silvio E. Inzucchi, Tzvetana Katova, Lars Køber, Mikhail N. Kosiborod, Anna Maria Langkilde, Daniel Lindholm, Charlotta E.A. Ljungman, Felipe A. Martinez, Eileen O’Meara, Marc S. Sabatine, Mikaela Sjöstrand, Scott D. Solomon, Sergey Tereshchenko, Subodh Verma, Pardeep S. Jhund, John J.V. McMurray

**Affiliations:** 1 BHF Cardiovascular Research Centre, University of Glasgow, UK (A.M.J., P.D., K.F.D., P.S.J., J.J.V.M.); 2 Department of Cardiology, University of Minnesota, Minneapolis (I.S.A.).; 3 2nd Department of Internal Medicine, Cardiovascular Medicine, General Teaching Hospital and 1st Faculty of Medicine, Charles University, Prague, Czech Republic (J.B.).; 4 Late Stage Development, Cardiovascular, Renal and Metabolism, BioPharmaceuticals R&D (O.B., A.M.L., D.L., M.S.), AstraZeneca, Gothenburg, Sweden.; 5 Cardiovascular, Renal and Metabolism Translational Medicines Unit, Early Clinical Development, IMED Biotech Unit (P.J.G.), AstraZeneca, Gothenburg, Sweden.; 6 Department of Cardiology, University Medical Center and University of Groningen, The Netherlands (R.A.d.B.).; 7 Department of Medicine, Saarland University Hospital, Homburg–Saar, Germany (M.B.).; 8 Quantitative Clinical Pharmacology, IMED Biotech Unit, Astra-Zeneca, Gaithersburg, MD (D.W.B.).; 9 Department of Cardiology, Medanta, Gurgaon, India (V.K.C.).; 10 Department of Biostatistics and Medical Informatics, University of Wisconsin, Madison (D.L.D.).; 11 5th Department of Internal Medicine, Comenius University in Bratislava, Slovakia (A.D.).; 12 Cumming School of Medicine and Libin Cardiovascular Institute, University of Calgary, AB, Canada (J.G.W.).; 13 Section of Endocrinology, Yale University School of Medicine, New Haven, CT (S.E.I.).; 14 Clinic of Cardiology, National Cardiology Hospital, Sofia, Bulgaria (T.K.).; 15 Rigshospitalet Copenhagen University Hospital, Denmark (L.K.).; 16 Saint Luke’s Mid America Heart Institute, University of Missouri, Kansas City (M.N.K.).; 17 Department of Molecular and Clinical Medicine and Cardiology, Sahlgrenska Academy, Gothenburg, Sweden (C.E.A.L.).; 18 National University of Cordoba, Argentina (F.A.M.).; 19 Department of Cardiology, Montreal Heart Institute, QC, Canada (E.O.).; 20 Division of Cardiovascular Medicine, Brigham and Women’s Hospital and Harvard Medical School, Boston, MA (M.S.S., S.D.S.).; 21 Department of Myocardial Disease and Heart Failure, National Medical Research Center of Cardiology, Moscow, Russia (S.T.).; 22 Division of Cardiac Surgery, St. Michael’s Hospital, University of Toronto, ON, Canada (S.V.).

**Keywords:** diuretics, heart failure, sodium-glucose transporter 2 inhibitors

## Abstract

In the DAPA-HF trial (Dapagliflozin and Prevention of Adverse-Outcomes in Heart Failure), the sodium-glucose cotransporter 2 inhibitor dapagliflozin reduced the risk of worsening heart failure and death in patients with heart failure and reduced ejection fraction. We examined the efficacy and tolerability of dapagliflozin in relation to background diuretic treatment and change in diuretic therapy after randomization to dapagliflozin or placebo.

**Methods:**

We examined the effects of study treatment in the following subgroups: no diuretic and diuretic dose equivalent to furosemide <40, 40, and >40 mg daily at baseline. We examined the primary composite end point of cardiovascular death or a worsening heart failure event and its components, all-cause death and symptoms.

**Results:**

Of 4616 analyzable patients, 736 (15.9%) were on no diuretic, 1311 (28.4%) were on <40 mg, 1365 (29.6%) were on 40 mg, and 1204 (26.1%) were taking >40 mg. Compared with placebo, dapagliflozin reduced the risk of the primary end point across each of these subgroups: hazard ratios were 0.57 (95% CI, 0.36–0.92), 0.83 (95% CI, 0.63–1.10), 0.77 (95% CI, 0.60–0.99), and 0.78 (95% CI, 0.63–0.97), respectively (*P* for interaction=0.61). The hazard ratio in patients taking any diuretic was 0.78 (95% CI, 0.68–0.90). Improvements in symptoms and treatment toleration were consistent across the diuretic subgroups. Diuretic dose did not change in most patients during follow-up, and mean diuretic dose did not differ between the dapagliflozin and placebo groups after randomization.

**Conclusions:**

The efficacy and safety of dapagliflozin were consistent across the diuretic subgroups examined in DAPA-HF.

**Registration:**

URL: https://www.clinicaltrials.gov; Unique identifier: NCT03036124.

## Abstract 2

Supplemental Digital Content is available in the text.

**Clinical Perspective**

**What Is New?**

- Inhibition of the sodium-glucose cotransporter 2 in the proximal renal tubule results in natriuresis, increased urinary glucose, and water loss.
- In the DAPA-HF trial (Dapagliflozin and Prevention of Adverse-Outcomes in Heart Failure), 84% of patients randomized were treated with a conventional diuretic such as a loop or thiazide diuretic.
- Little is known about the safety and efficacy of combining treatment with a sodium-glucose cotransporter 2 inhibitor and conventional diuretics in patients with heart failure and reduced ejection fraction.

**What Are the Clinical Implications?**

- Our findings show that treatment with dapagliflozin is safe and effective regardless of diuretic use or diuretic dose.
- The majority of patients in the DAPA-HF trial did not change their diuretic dose throughout follow-up, and although a decrease in diuretic dose was more frequent with dapagliflozin than with placebo, the between-group difference was small.
- There was an elevation in hematocrit that persisted regardless of baseline diuretic dose and despite a reduction in diuretic dose during follow-up, suggesting that mechanisms other than hemoconcentration might, in part, account for this observation.

**Editorial, see p 1055**

Because sodium-glucose cotransporter 2 (SGLT2) reabsorbs sodium, in addition to glucose, in the proximal renal tubule, SGLT2 inhibition leads to natriuresis, as well as an increase in urinary glucose and accompanying water excretion.^1^ Consequently, SGLT2 inhibitors have a diuretic action in healthy volunteers and in individuals with type 2 diabetes mellitus. Clearly, a diuretic action might be beneficial in patients with heart failure and reduced ejection fraction (HFrEF) but could also be a double-edged sword. Patients with HFrEF are usually treated with a conventional diuretic, most often a loop diuretic. However, little is known about the effects of adding an SGLT2 inhibitor in people treated with conventional diuretic therapy. It is possible that SGLT2 inhibitors might augment the effect of conventional diuretics, with attendant risk of volume depletion. Conversely, because the diuretic effect of SGLT2 inhibitors is modest, they may add little, if anything, to the potent effect of loop diuretics (and the combination of a loop diuretic and a mineralocorticoid receptor antagonist [MRA]). Alternatively, an intermediate possibility is that SGLT2 inhibitors might have a diuretic-sparing effect in patients with HFrEF.

Kidney function is clearly another key consideration in patients with HFrEF, many of whom have renal impairment. If there is significant augmentation of diuresis resulting from the addition of an SGLT2 inhibitor to conventional therapy, this might lead to worsening kidney function through volume depletion and decreased renal perfusion, especially in the setting of renin-angiotensin-aldosterone system blockade. Conversely, because the glycosuric effect of SGLT2 inhibitors diminishes as renal function declines, so might the diuretic action of these agents, rendering them less effective in patients with HFrEF with chronic kidney disease.^1^

Therefore, understanding the interaction between the effects of SGLT2 inhibitors and diuretic therapy is of fundamental importance to the use of SGLT2 inhibitors in HFrEF. The DAPA-HF trial (Dapagliflozin and Prevention of Adverse-Outcomes in Heart Failure) demonstrated that SGLT2 inhibition reduced the risk of worsening HF or death resulting from cardiovascular causes compared with placebo in patients with HFrEF.^2^ In DAPA-HF, 84% of patients were prescribed a diuretic at baseline in addition to other standard therapy, including a renin-angiotensin inhibitor in 94% and an MRA in 71%.^2^ We examined the effect of dapagliflozin compared with placebo on efficacy and safety outcomes according to baseline diuretic therapy and on change in diuretic requirement and possible markers of volume status over time.

## Methods

### Study Design and Criteria

The design and primary results of DAPA-HF have been published.^2,3^ The Ethics Committee for each participating institution approved the protocol, and all patients gave written informed consent. Eligibility for the trial included New York Heart Association class II to IV symptoms, an ejection fraction of ≤40%, and an elevated NT-proBNP (N-terminal B-type natriuretic peptide) level. For patients who had been hospitalized within the preceding 12 months, the NT-proBNP threshold was ≥400 pg/mL; for any patient with atrial fibrillation or flutter, the threshold was ≥900 pg/mL; and for patients who met neither of these criteria, the threshold was ≥600 pg/mL. Investigators were asked to ensure that patients were optimally treated with pharmacological and device therapy for HFrEF, in keeping with local guidelines. The protocol advised that an angiotensin-converting enzyme inhibitor, angiotensin receptor blocker, or sacubitril/valsartan and a β-blocker, as well as an MRA, should be used at guideline-recommended doses unless contraindicated or not tolerated. The protocol also stated that in patients taking diuretics, the goal of treatment was achieving optimal fluid volume status in each individual. The main exclusion criteria included recent treatment with an SGLT2 inhibitor, symptomatic hypotension or a systolic blood pressure of <95 mm Hg, an estimated glomerular filtration rate of <30 mL·min^−1^·1.73 m^−2^, rapidly declining renal function, or type 1 diabetes mellitus.

Data underlying the findings described in this article may be obtained in accordance with AstraZeneca’s data-sharing policy.

### Trial Procedures

After a 14-day screening period, patients were randomized in a 1:1 fashion to receive dapagliflozin at a dose of 10 mg once daily or placebo. Randomization was stratified according to diabetes mellitus status (either a history of diabetes mellitus or a glycated hemoglobin level of ≥6.5% at screening). Patients were evaluated after randomization at 14 days, 2 and 4 months, and every 4 months thereafter.

### Trial Outcomes

The primary outcome was the composite of an episode of worsening HF or cardiovascular death, whichever occurred first. An episode of worsening HF was either an unplanned hospitalization or an urgent visit resulting in intravenous therapy for HF. Secondary outcomes included change in Kansas City Cardiomyopathy Questionnaire total symptom score from baseline to 8 months (a lower score equates to worse symptoms) and a composite of worsening renal function and death resulting from any cause. Safety outcomes included all serious adverse events and specific adverse events, including those associated with discontinuation of the study drug and other events of interest such as volume depletion and renal events. Changes in laboratory and clinical findings from baseline to 8 months were also examined.

### Statistical Analysis

We included patients treated and not treated with a diuretic at baseline. Patients treated with a diuretic were included if they had information on dose. MRAs were not classed as a diuretic for the purposes of this analysis. Bumetanide 1 mg, torsemide 20 mg, azosemide 60 mg, and etacrynic acid 100 mg were considered equivalent to furosemide 40 mg intravenously and furosemide 80 mg orally. If at any time the loop diuretic dose was indeterminate, the patient was excluded from the analyses at that time point only and included again as soon as the dose could be determined. If a patient was not taking a loop diuretic, the patient was classified as being on 0 mg until he or she either was started on a loop diuretic or died (at which point the dose was recorded as missing). The following furosemide-equivalent oral daily dose categories were generated: <40 mg, 40 mg exactly, and >40 mg, in addition to a “no diuretic” category. Patients on a nonloop diuretic only (eg, a thiazide diuretic alone) were included in the group of <40 mg of furosemide-equivalent loop diuretic. Only a small number of patients were taking a combination (mostly a loop and thiazide or thiazide-like diuretic; see Results).

Baseline characteristics were compared across diuretic therapy categories with the use of ANOVA, Kruskal-Wallis tests, and χ^2^ tests. Proportional changes in diuretic dose from baseline to 14 days and to 2, 6, 12, and 18 months were modeled with logistic regression. As a sensitivity analysis, a partial proportional odds model was also used to examine change in diuretic dose at different time points.

Changes in hematocrit, creatinine, NT-proBNP, systolic blood pressure, and weight were analyzed with a mixed model for repeated measures (adjusted for baseline values, randomized treatment, and interaction of treatment and visit, with a random intercept and slope per patient), and the between-treatment-group differences at 8 months after randomization were presented by diuretic subgroup as least-square-means difference and 95% CI. Because improvement in clinical status with any treatment for HFrEF may reduce diuretic requirement, for comparison, we also examined change in diuretic dose over time in the CHARM trials (Candesartan in Heart Failure Assessment of Reduction in Mortality and Morbidity) of low left ventricular ejection fraction.^4^

Time-to-first-event outcomes (the primary composite outcome and its components, hospitalization for heart failure/urgent heart failure visit or cardiovascular death, worsening renal function, and death resulting from any cause) were analyzed with Cox regression. Cumulative first events were displayed with Kaplan-Meier curves. The interaction between diuretic therapy and the effect of treatment was assessed with the respective statistical model for that measure or outcome and with a logistic regression model for the safety outcomes. All models were stratified by or adjusted for diabetes mellitus status and, except in the cases of death resulting from any cause and the composite worsening renal function end point (the latter included baseline estimated glomerular filtration rate), previous hospitalization for heart failure. Additional variables included in adjusted models were age, sex, race, New York Heart Association class, left ventricular ejection fraction, cause of heart failure, NT-proBNP (log), heart rate, systolic blood pressure, estimated glomerular filtration rate, atrial fibrillation/flutter, and previous hospitalization for heart failure if not already included in the model (except for death resulting from any cause). All analyses were conducted with STATA version 16 (StataCorp LLC, College Station, TX). A value of *P*<0.05 was considered statistically significant.

## Results

Of the 4744 patients randomized, 736 (15.5%) were not taking a diuretic, and of the remaining 4008 participants, 3880 had a determinable diuretic dose (expressed as the furosemide-equivalent dose). Of those receiving a diuretic (n=3880), the largest number (1365 patients; 29.6% of all analyzed patients and 35.2% of patients receiving a diuretic) were taking a furosemide-equivalent dose of 40 mg (Table 1). The number taking >40 mg was 1204 (26.1% and 31.0%, respectively), and the number taking <40 mg or a nonloop diuretic only was 1311 (28.4% and 33.8%, respectively). The most commonly used loop diuretic among those prescribed a loop diuretic was furosemide. Bumetanide was used more frequently in patients on a dose of >40 mg furosemide equivalent than in patients on lower doses of loop diuretic. Overall, 320 (6.9% and 8.2%, respectively) were taking a combination of diuretics; in most cases (272, 85.0%), this was the combination of a loop and thiazide or thiazide-like diuretic alone. A similar proportion of patients in each diuretic group were randomized to dapagliflozin or placebo.

**Table 1.**
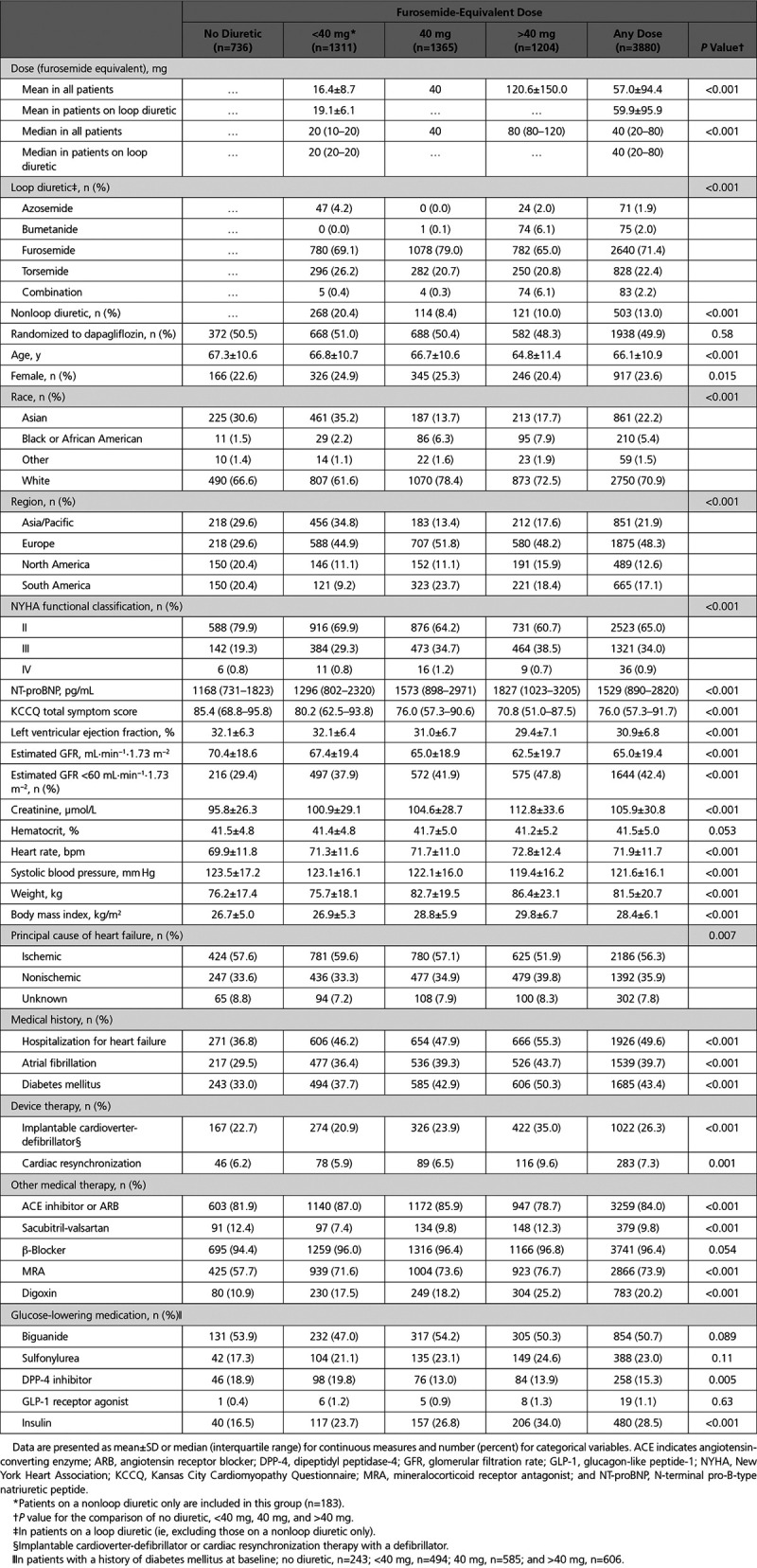
Baseline Characteristics According to Diuretic Therapy at Baseline

### Patient Characteristics

There were several differences between patients in the different diuretic groups (Table 1). Compared with those not receiving a diuretic, patients in the highest diuretic dose category (>40 mg) were younger; had worse symptoms, functional class, and renal function; had higher NT-proBNP levels and body mass index and lower left ventricular ejection fraction and systolic blood pressure; and were more likely to have a history of heart failure hospitalization, atrial fibrillation, and diabetes mellitus. Hematocrit was similar between groups at baseline. Patients in the highest diuretic dose category at baseline were more often treated with an MRA and digoxin. The small group (n=320) of patients taking the combination of a loop and another diuretic had a more severe profile overall (Table I in the Data Supplement).

### Change in Diuretic Dose After Randomization

**Figure 1.**
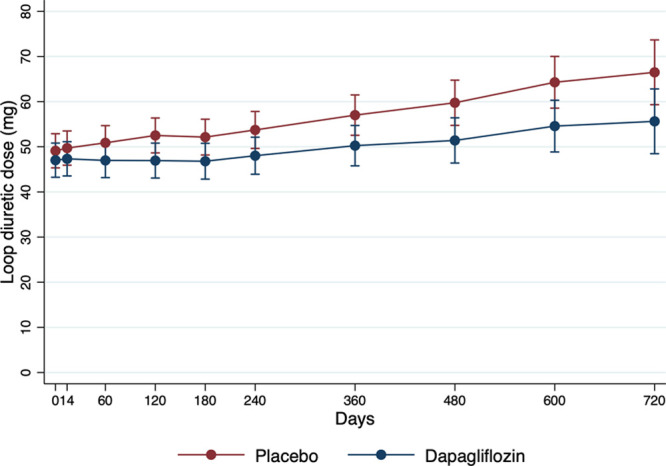
**Change in loop diuretic dose over time in all patients.**

The mean (SD) furosemide-equivalent dose in patients taking any diuretic was 57.0 (94.4) mg; in patients taking a loop diuretic, it was 59.9 (95.9) mg, with no statistically significant difference between treatment groups (Table 2). There was no change in the loop diuretic dose in most patients at 2 weeks and at 2, 6, 12, and 18 months (97.1%, 91.4%, 83.3%, 77.2%, and 73.6%, respectively); this was the case in patients randomized to placebo or dapagliflozin. Throughout follow-up, the mean dose of loop diuretic rose at a similar rate in both treatment arms (Figure 1), and this was the case regardless of baseline dose (Figure I in the Data Supplement). The findings were very similar in CHARM (Figure II in the Data Supplement).

**Table 2.**
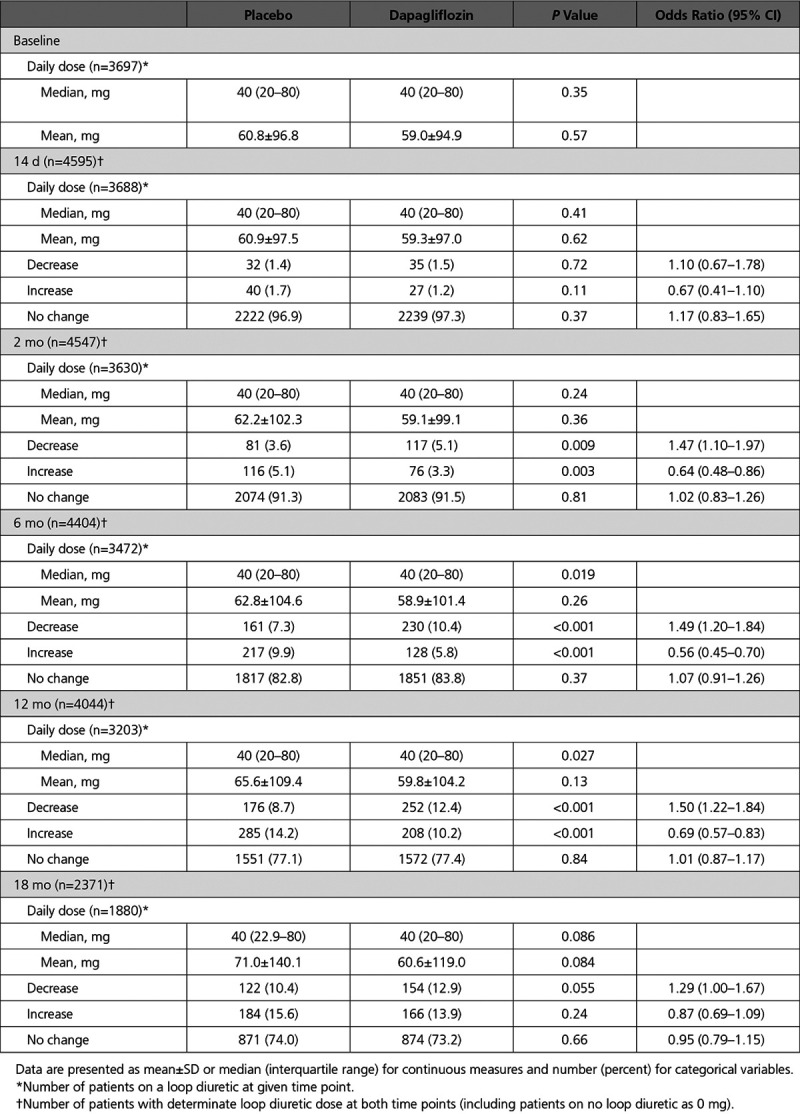
Loop Diuretic Doses and Changes in Loop Diuretic Dose From Baseline

A small proportion of patients had their dose of diuretic reduced, and this happened more frequently with dapagliflozin than with placebo (at 6 months, 10.4% versus 7.3%, respectively, *P*<0.001; at 12 months, 12.4% versus 8.7%, *P*<0.001). Conversely, fewer patients in the dapagliflozin arm compared with the placebo arm had an increase in diuretic dose during follow-up, although again the proportion of patients in either treatment arm with an increase in diuretic dose was small (at 6 months, 5.8% versus 9.9%, *P*<0.001; at 12 months, 10.2% versus 14.2%, *P*<0.001). The likelihood of a change in diuretic dose was similar whether a logistic regression or a proportional odds model was used. In comparison, the findings were very similar in CHARM (Table II in the Data Supplement).

After an initial change in dose of diuretic, further changes tended to reverse the original change; for example, when the initial change was an increase in dose, the subsequent change was more likely to be a decrease (Figure III in the Data Supplement).

### Clinical Outcomes According to Baseline Diuretic Treatment

The cumulative incidences of the primary composite outcome, hospitalization or urgent visit for heart failure, death resulting from cardiovascular causes, and death resulting from any cause were all lowest in patients not taking any diuretic and highest in those receiving a furosemide-equivalent dose of >40 mg (Figure IV in the Data Supplement).

**Figure 2.**
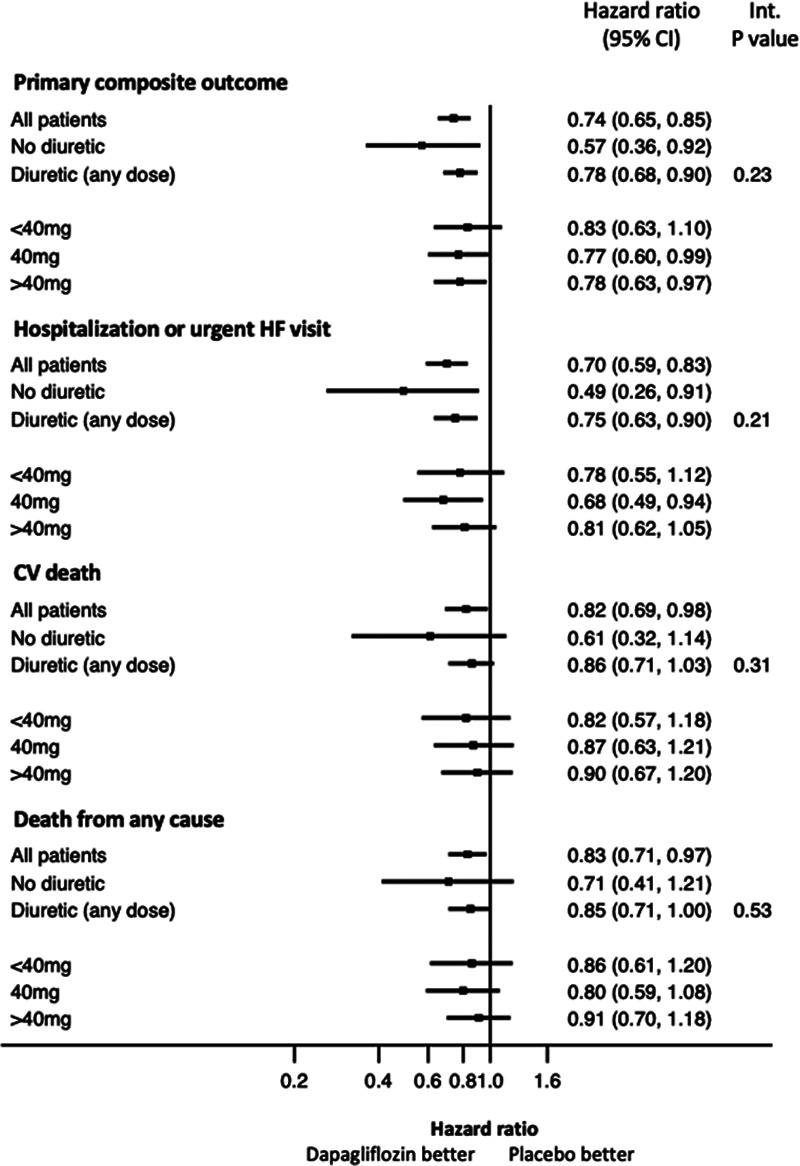
**Forest plot of efficacy outcomes according to diuretic therapy at baseline.**

The risks of the primary composite outcome and its components (hospitalization or urgent visit for heart failure and death resulting from cardiovascular causes) were reduced by dapagliflozin compared with placebo in all patients, whether treated or not treated with a diuretic, and regardless of dose in patients receiving a diuretic (Table 3 and Figure 2). The single possible exception to this was in the group of patients taking a combination of a loop and another diuretic at baseline, although the number of events in this group was small, the 95% CI around the hazard ratio was wide, and the interaction *P* value was nonsignificant (Table I in the Data Supplement). Findings for the components of the primary end point and the other mortality/morbidity outcomes were consistent.

**Table 3.**
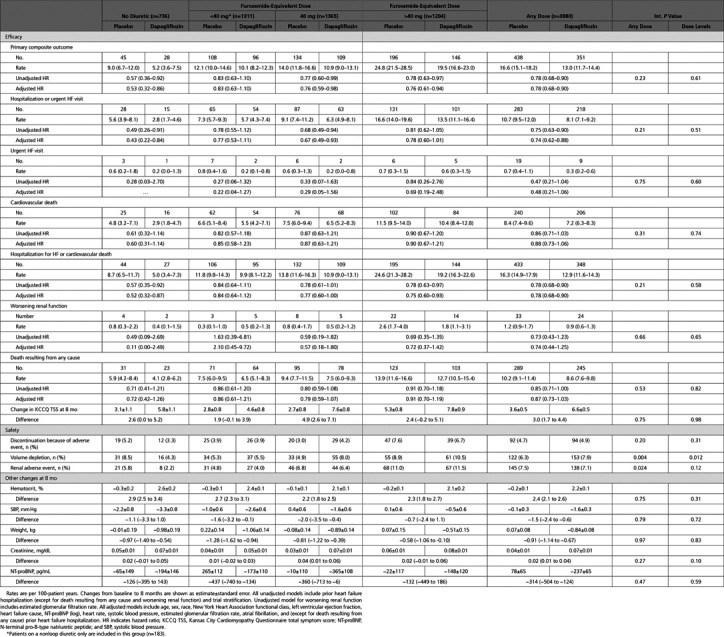
Treatment Effect on Outcomes, Safety, and Events of Interest According to Diuretic Therapy at Baseline

When the effect of randomized treatment was examined as a function of change in diuretic dose from baseline to 6 months (decrease, increase, or no change), the incidence of the primary composite outcome was lower with dapagliflozin compared with placebo in each diuretic dose change group (*P* for interaction=0.27; Figure V in the Data Supplement).

The placebo-corrected improvement in Kansas City Cardiomyopathy Questionnaire total symptom score was consistent in patients treated with dapagliflozin, regardless of diuretic treatment or no diuretic treatment or diuretic dose (Table 3).

Because the number of patients experiencing the prespecified renal composite outcome was small overall, meaningful interpretation of the subgroups was difficult. However, the rate of this outcome was highest in the patients on the highest diuretic dose and did not differ between dapagliflozin and placebo in that diuretic dose group.

### Safety

The frequency of volume depletion, renal adverse events, and study drug discontinuation as a result of an adverse event varied according to background diuretic therapy in the placebo arm. The rates of all adverse outcomes of interest were lower in patients not treated with a diuretic at baseline compared with patients treated with a diuretic. Among patients treated with a diuretic at baseline, the placebo rate of each adverse outcome was highest in patients on a dose of >40 mg furosemide equivalent (Table 3).

When the randomized treatment arms were compared, volume depletion and renal adverse events were significantly less common in patients allocated to dapagliflozin compared with those randomized to placebo in patients not taking diuretics at baseline. In patients taking diuretics, volume depletion was slightly more common with dapagliflozin than with placebo in patients in the higher-dose diuretic groups. Other adverse effects were not different, and there was no difference between treatment groups in study drug discontinuation for adverse events.

### Other Measures of Interest

**Figure 3.**
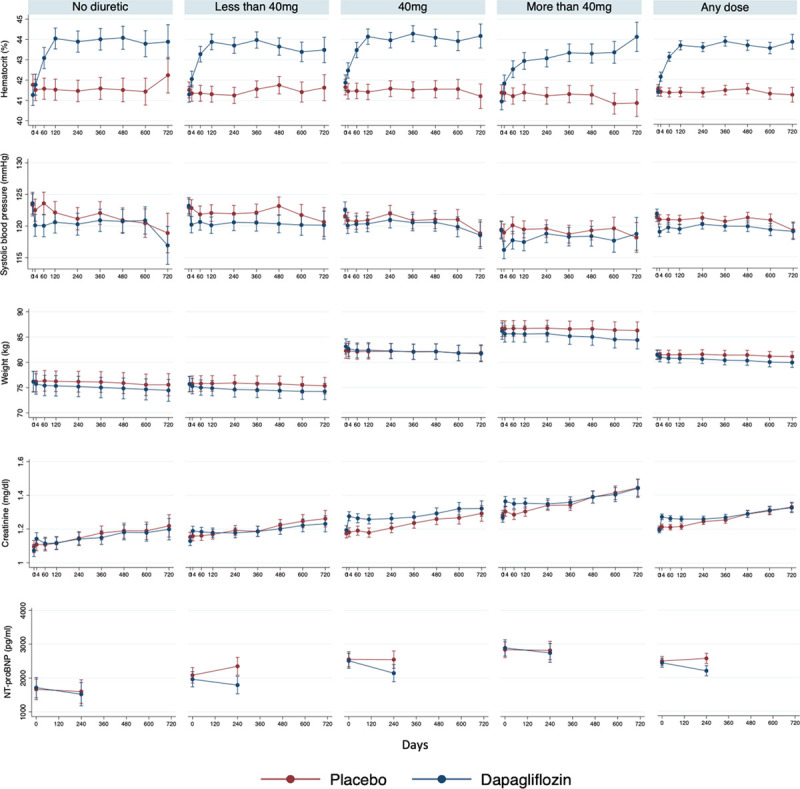
**Changes in laboratory and clinical measures according to diuretic therapy at baseline.**

From baseline to 8 months, the placebo-corrected increases in hematocrit and creatinine and decreases in systolic blood pressure and weight with dapagliflozin were similar in patients receiving and those not receiving diuretics at baseline (Table 3 and Figure 3). Although the decrease in NT-proBNP was numerically greater in patients receiving diuretics, the variance in this measure was large, and the difference was not statistically significant.

In a comparison across the diuretic dose groups, none of the placebo-corrected changes with dapagliflozin differed significantly, although the changes tended to be smallest in the highest diuretic dose group.

The elevation in hematocrit with dapagliflozin was sustained, regardless of dose of loop diuretic (Table 3 and Figure 3). This persisted regardless of whether the dose of conventional diuretic was decreased, was increased, or remained the same at 6 and 12 months (Figure VI in the Data Supplement).

## Discussion

The key finding of the present analyses is that the benefits of dapagliflozin in DAPA-HF were obtained regardless of the use of background diuretic therapy or dose of diuretic therapy. Likewise, the tolerability and safety profile of dapagliflozin compared with placebo was similar regardless of concomitant treatment with a conventional diuretic or dose of conventional diuretic. Last, most patients did not have a change in dose of diuretic during follow-up, and the mean daily dose of diuretic did not differ between the dapagliflozin and placebo groups.

The use of diuretic therapy at baseline in DAPA-HF was in keeping with other reports in large cohorts of patients with HFrEF, predominantly in New York Heart Association class II and III. In DAPA-HF, 16% of patients were not treated with a diuretic at baseline (not counting an MRA as a diuretic), a proportion consistent with that reported in other recent trials and registries.^5–10^

Among all patients randomized in DAPA-HF, 81% were treated with a loop diuretic, also in keeping with other studies.^11–15^ In DAPA-HF, the mean and median furosemide-equivalent doses were 60 and 40 mg, respectively, with the largest proportion receiving a daily dose of 40 mg (35.2% of patients taking any diuretic). In PARADIGM-HF (Prospective Comparison of ARNI [Angiotensin Receptor Neprilysin Inhibitor] With ACEI to Determine Impact on Global Mortality and Morbidity in Heart Failure), the mean daily dose was 49 mg, and 60.6% of patients were treated with between 20 and 40 mg at baseline, although the dose conversion calculation was different in this study.^11^ Additional information on loop diuretic dosing is hard to obtain; however, in the CHARM HFrEF trials, in the recent HFrEF registry from the Netherlands, and in the HF-ACTION trial (Heart Failure: A Controlled Trial Investigating Outcomes of Exercise Training), the median dose was also 40 mg.^4,12,15^

Despite notable differences in patient characteristics according to use of diuretic at baseline (and according to dose of diuretic among those taking diuretics), the benefit of dapagliflozin was consistent in all the diuretic groups studied. As expected, patients not receiving diuretics had a more favorable clinical profile and were at lowest risk of the clinical outcomes evaluated. The converse was true of patients in the highest diuretic dose category. Although the magnitude of the benefit from dapagliflozin appeared to be larger in patients not treated with a diuretic, there were relatively few patients in this group, and the CIs around the point estimate for the effect of treatment were wide. A statistical test for interaction between background diuretic therapy and the effect of randomized treatment was not significant. In addition, there was no suggestion of any dose response in relation to baseline diuretic therapy, and diuretic dose group did not modify the effect of randomized treatment in an interaction test. Consistent with this, the effect of dapagliflozin on other measures such as systolic blood pressure, weight, creatinine, and hematocrit did not vary substantially between patients treated and those not treated with a diuretic and across the diuretic dose groups.

The tolerability and safety of dapagliflozin were also consistent in patients treated and those not treated with diuretics and across the diuretic dose groups studied. In this analysis, our focus was on the prespecified adverse events related to volume depletion and renal dysfunction. Adverse events related to volume depletion were relatively infrequent overall but, as expected, were more common in patients receiving higher doses of diuretic (ranging from 5.3% to 8.9% in the placebo arm, from the lowest to highest diuretic dose group). These events were numerically slightly more common in patients randomly assigned to dapagliflozin compared with those assigned to placebo, although the excess was small (0.2% in the lowest diuretic dose group, 3.1% in the middle dose group, and 1.6% in the highest dose group). More surprisingly, the rate of adverse events related to volume depletion was as high in the patients not receiving diuretic at baseline as in patients in the highest diuretic dose group (8.5% and 8.9% in the placebo arms, respectively). In patients not receiving diuretic at baseline, the rate of adverse events related to volume depletion after randomization was lower in patients assigned to dapagliflozin than in those assigned to placebo. Similar patterns were seen for renal adverse events in the placebo group in relation to baseline diuretic therapy (more common with increasing diuretic dose, more common in patients not taking diuretics than in those in the lowest diuretic dose group in the placebo arm). However, renal adverse events were generally less common in patients assigned to dapagliflozin compared with those assigned to placebo, a pattern opposite that observed for volume depletion. Few patients stopped study drug for an adverse event, generally <5% in any subgroup except patients taking >40 mg/d furosemide or equivalent, and in that subgroup, the proportion was 7.6% in the placebo group and 6.7% in the dapagliflozin group.

Although our findings go some way to assuaging the hypothetical safety concerns about combining SGLT2 inhibitors with conventional diuretics (and MRAs) in HFrEF, they do not directly address the reported diuretic action of SGLT2 inhibitors. However, our analyses provide evidence that the combination of dapagliflozin with conventional diuretics in DAPA-HF did not have a major impact on the use of loop diuretics in the majority of patients. The mean dose of furosemide did not differ between the dapagliflozin and placebo group during follow-up, and most patients did not change their diuretic dose. A small proportion did have an increase or decrease in dose, and an increase was less likely and a decrease was more likely in the dapagliflozin arm compared with the placebo arm (between-treatment difference in proportion was <5% at all time points examined). Moreover, this small difference was similar to that seen with other treatments that improve symptoms and reduce worsening of heart failure status over time but do not have diuretic properties, as illustrated in the comparison with CHARM and in a report from the PARADIGM-HF trial.^11^ These findings do not preclude a diuretic effect of dapagliflozin (and clinical benefit related to such an action), and previous studies have shown a short-lived initial natriuresis with SGLT2 inhibitors with restoration of a new sodium volume steady state within days to weeks of treatment starting.^16–19^ In addition, the increased electrolyte-free water excretion induced by SGLT2 inhibitors and possible enhanced peripheral sodium storage are other factors that may contribute to the impact of these agents on whole-body sodium volume status.^20,21^ However, the similar effects of dapagliflozin in patients with chronic kidney disease and in those without diabetes mellitus, 2 groups who should have had less diuretic effect from SGLT2 inhibition, argue for additional mechanisms of benefit.^2^ Although the increase in hematocrit seen with SGLT2 inhibitors is also often said to reflect a diuretic-induced reduction in blood volume, this assumption may be overly simplistic. The increase in hematocrit in DAPA-HF was similar, regardless of use of diuretic at baseline or baseline loop diuretic dose, and was seen despite rates of volume depletion similar to those observed in the placebo group. The elevation in hematocrit also persisted in individuals with a reduction in loop diuretic dose at both 6 and 12 months. Collectively, these findings support the notion that factors other than hemoconcentration such as augmentation of erythropoiesis may account for the sustained increase in hematocrit seen with dapagliflozin. A reduction in circulating levels of hepcidin, an increase in levels of the hepcidin inhibitor erythroferrone, and an increase in erythropoietin have been shown to occur with the use of SGLT2 inhibitors.^22–24^

The study has some limitations. This was not a prespecified analysis. Furosemide-equivalent loop diuretic doses were not available for all patients. Dose changes were examined at specified time points, and fluctuations in doses in between these time points were not accounted for in this analysis. We did not have data on other markers of natriuresis and diuresis such as urine volumes and urinary sodium excretion. Our results apply only to ambulatory patients with HFrEF predominantly in New York Heart Association classes II and III who fulfilled the trial inclusion and exclusion criteria and received modest doses of conventional diuretic therapy. We do not know what the efficacy and safety of dapagliflozin might be in different patients receiving a different treatment in a different setting.

### Conclusions

We found that the benefits of dapagliflozin were obtained regardless of background diuretic therapy and across the range of background doses of diuretic used in DAPA-HF. The tolerability and safety of dapagliflozin were similar regardless of whether patients were treated or not treated with a standard diuretic and dose of conventional diuretic. Treatment with dapagliflozin did not lead to a change in mean dose of background diuretic therapy in most trial participants.

## Sources of Funding

Dr Jackson is supported by a British Heart Foundation Clinical Research Training Fellowship (FS/18/14/33330), and Dr McMurray is supported by a British Heart Foundation Centre of Research Excellence Grant (RE/18/6/34217).

## Disclosures

Dr Anand reports receiving fees for serving as US national leader of a trial from AstraZeneca; fees for serving on a steering committee from ARCA Biopharma, Amgen, LivaNova, and Novartis; fees for serving on an end-point committee from Boehringer Ingelheim; fees for serving as chair of a data and safety monitoring board from Boston Scientific; and advisory board fees from Zensun. Dr Bělohlávek reports receiving advisory board fees from Novartis and Pfizer and lecture fees from Getginge. Drs Bengtsson, Greasley, Lindholm, and Sjöstrand report being employed by AstraZeneca. Dr de Boer has received grant support (paid to University Medical Center Groningen [UMCG]), consulting fees, and lecture fees from AstraZeneca, grant support (paid to UMCG) from Bristol-Myers Squibb, grant support (paid to UMCG) and consulting fees from Abbott, grant support (paid to UMCG) and lecture fees from Roche, and consulting fees from MandalMed and is a minority shareholder in scPharmaceuticals. Dr Böhm reports receiving lecture fees from Amgen, Bayer, Servier, Medtronic, Boehringer Ingelheim, Vifor Pharma, and Bristol-Myers Squibb; grant support and lecture fees from AstraZeneca; and grant support from Deutsche Forschungsgemeinschaft. Dr Boulton has received personal fees from AstraZeneca. Dr DeMets has received consulting fees from Frontier Science, Actelion, Bristol-Myers Squibb, Medtronic, Boston Scientific, GlaxoSmithKline, and Merck and has received consulting fees from and is owner of DL DeMets Consulting. Dr. Docherty has received grant support from Novartis. Dr Howlett reports receiving grant support, consulting fees, and lecture fees from AstraZeneca, Boehringer Ingelheim, Novartis, and Servier; consulting fees and lecture fees from Novo Nordisk; consulting fees from Janssen; and grant support, consulting fees, lecture fees, and provision of drugs from Pfizer. Dr Inzucchi reports receiving advisory fees from AstraZeneca and Zafgen; lecture fees, consulting fees, fees for serving as a clinical trial publications committee member, reimbursement for medical writing, and travel support from Boehringer Ingelheim; fees for serving on a steering committee and travel support from Sanofi-Lexicon; lecture fees, consulting fees, and travel support from Merck; and advisory fees and travel support from vTv Therapeutics and Abbott-Alere. Dr Katova reports receiving fees for serving as national coordinator of a trial from Novartis and AstraZeneca. Dr. Køber has received lecture fees from Novartis and Bristol-Myers Squibb. Dr Kosiborod has received grant support, honoraria, and research support from AstraZeneca; grant support and honoraria from Boehringer Ingelheim; and honoraria from Sanofi, Amgen, Novo Nordisk, Merck (Diabetes), Eisai, Janssen, Bayer, GlaxoSmithKline, Glytec, Intarcia Therapeutics, Novartis, Applied Therapeutics, Amarin, and Eli Lilly. Dr Langkilde reports being employed by and holding shares in AstraZeneca. Dr. Ljungman reports receiving lecture fees and advisory board fees from AstraZeneca, lecture fees from Novartis, and advisory board fees from Pfizer. Dr Martinez has received personal fees from AstraZeneca as honoraria. Dr O’Meara has received fees for serving on a clinical trial (paid to her institution), consulting fees, and lecture fees from AstraZeneca, Bayer, Amgen, and Novartis; consulting fees from Merck; fees for serving on a clinical trial (paid to her institution) from American Regent; and consulting fees and lecture fees from Pfizer and Boehringer Ingelheim. Dr Sabatine reports receiving grant support (paid to Brigham and Women’s Hospital) and consulting fees from Amgen, AstraZeneca, Intarcia Therapeutics, Janssen Research and Development, The Medicines Company, MedImmune, Merck, and Novartis; receiving consulting fees from Anthos Therapeutics, Bristol-Myers Squibb, CVS Caremark, DalCor Pharmaceuticals, Dyrnamix, Esperion, IFM Therapeutics, and Ionis Pharmaceuticals; receiving grant support (paid to Brigham and Woman’s Hospital) from Bayer, Daiichi Sankyo, Eisai, GlaxoSmithKline, Pfizer, Poxel, Quark Pharmaceuticals, and Takeda Pharmaceutical; and serving as a member of the TIMI (Thrombolysis in Myocardial Infarction) Study Group, which receives grant support (paid to Brigham and Women’s Hospital) from Abbott, Aralez Pharmaceuticals, Roche, and Zora Biosciences. Dr Solomon reports receiving grant support and consulting fees (all fees listed paid to Brigham and Women’s Hospital) from Alnylam Pharmaceuticals, Amgen, AstraZeneca, Bristol-Myers Squibb, Gilead Sciences, GlaxoSmithKline, MyoKardia, Novartis, Theracos, Bayer, and Cytokinetics; grant support from Bellerophon Therapeutics, Celladon, Ionis Pharmaceuticals, Lonestar Heart, Mesoblast, Sanofi Pasteur, and Eidos Therapeutics; consulting fees from Akros Pharma, Corvia Medical, Ironwood Pharma, Merck, Roche, Takeda Pharmaceutical, Quantum Genomics, AOBiome, Cardiac Dimensions, Tenaya Therapeutics, and Daiichi Sankyo; and fees for serving on a data and safety monitoring board from Janssen. Dr Tereshchenko reports receiving lecture fees from Servier, Pfizer, Novartis, and Boehringer Ingelheim; Dr Verma reports receiving grant support, lecture fees, and advisory board fees from AstraZeneca, Boehringer Ingelheim, Bayer, Janssen, and Merck; lecture fees from Sun Pharmaceutical Industries and EOCI Pharmacomm; grant support and advisory board fees from Amgen; and lecture fees and advisory board fees from Sanofi and Eli Lilly. Dr. Jhund reports receiving consulting fees, advisory board fees, and lecture fees from Novartis; advisory board fees from Cytokinetics; and grant support from Boehringer Ingelheim. Dr. McMurray reports receiving fees (all fees listed paid to Glasgow University) for serving on a steering committee from Bayer; fees for serving on a steering committee, fees for serving on an end-point committee, and travel support from Cardiorentis; fees for serving on a steering committee and travel support from Amgen and Oxford University–Bayer; fees for serving as principal investigator of a trial and travel support from Theracos; fees for serving on a steering committee and travel support from AbbVie; fees for serving on a steering committee from DalCor Pharmaceuticals; fees for serving on a data and safety monitoring committee from Pfizer and Merck; fees for serving on an executive committee, fees for serving as co–principal investigator of a trial, fees for serving on a steering committee, fees for serving on an executive committee, travel support, and advisory board fees from Novartis; fees for serving as co–principal investigator for a trial, fees for serving on a steering committee, and travel support from GlaxoSmithKline; fees for serving on a steering committee from Bristol-Myers Squibb; and fees for serving on a steering committee, fees for serving on an end-point adjudication committee, and travel support from Vifor Pharma–Fresenius. The other authors report no conflicts.

## Supplemental Materials

Data Supplement Tables I and II

Data Supplement Figures I–VI

## Supplementary Material


