# Supplementary material for: Dapagliflozin and Diuretic Use in Patients With Heart Failure and Reduced Ejection Fraction in DAPA-HF
Source: Circulation. 2020 Jul 16;142(11):1040–54. doi: 10.1161/CIRCULATIONAHA.120.047077 (PMC7664959; doi:10.1161/CIRCULATIONAHA.120.047077)
Supplement: Supplementary file 1 [file cir-142-1040-s001.pdf]

SUPPLEMENTAL MATERIAL

Supplemental Table I. Baseline characteristics, efficacy and safety according to combinations of diuretic classes

	**No diuretic**<br><br>**(n=736)**	**Loop diuretic only**<br><br>**(n=3377)**	**Non-loop diuretic only**<br><br>**(n=183)**	**Loop and non-loop diuretic**<br>**(n=320)**	**P value**
Dose (furosemide-equivalent) – mg					
Mean in all patients	-	58.5±95.4	-	74.3±100.1	<0.001
Median in all patients	-	40 (20-80)	-	40 (35.1-80)	<0.001
Loop diuretic					0.042
Azosemide	-	60 (1.8)	-	11 (3.4)	
Bumetanide	-	69 (2.0)	-	6 (1.9)	
Furosemide	-	2428 (71.9)	-	212 (66.2)	
Torsemide	-	749 (22.2)	-	79 (24.7)	
Combination	-	71 (2.1)	-	12 (3.8)	
Non-loop diuretic					<0.001
Thiazide or thiazide-like	-	-	177 (96.7)	272 (85.0)	
Other	-	-	2 (1.1)	36 (11.2)	
Combination	-	-	4 (2.2)	12 (3.8)	
Randomized to dapagliflozin	372 (50.5)	1683 (49.8)	94 (51.4)	161 (50.3)	0.97
Age – years	67.3±10.6	66.0±10.9	68.1±10.5	66.2±11.1	0.002
Female	166 (22.6)	766 (22.7)	52 (28.4)	99 (30.9)	0.003
Race					<0.001
Asian	225 (30.6)	747 (22.1)	39 (21.3)	75 (23.4)	
Black or African American	11 (1.5)	184 (5.4)	5 (2.7)	21 (6.6)	
Other	10 (1.4)	48 (1.4)	7 (3.8)	4 (1.2)	
White	490 (66.6)	2398 (71.0)	132 (72.1)	220 (68.8)	
Region					<0.001
Asia/Pacific	218 (29.6)	738 (21.9)	38 (20.8)	75 (23.4)	

Europe	218 (29.6)		1627 (48.2)		75 (41.0)		173 (54.1)		
North America	150 (20.4)		444 (13.1)		19 (10.4)		26 (8.1)		
South America	150 (20.4)		568 (16.8)		51 (27.9)		46 (14.4)		
NYHA functional classification									<0.001
II	588 (79.9)		2195 (65.0)		146 (79.8)		182 (56.9)		
III	142 (19.3)		1147 (34.0)		37 (20.2)		137 (42.8)		
IV	6 (0.8)		35 (1.0)		0 (0.0)		1 (0.3)		
NT-proBNP – pg/ml	1168 (731-1823)		1541 (903-2815)		1034 (704-1908)		1824 (1005-3503)		<0.001
KCCQ total symptom score	85.4 (68.8-95.8)		76.0 (57.3-91.7)		83.3 (67.7-95.8)		70.8 (52.6-87.5)		<0.001
Left ventricular ejection fraction – %	32.1±6.3		30.7±6.9		33.3±6.0		31.9±6.7		<0.001
Estimated GFR – ml/min/1.73m^2	70.4±18.6		65.4±19.4		67.0±19.6		59.9±18.4		<0.001
Heart rate – bpm	69.9±11.8		71.9±11.6		71.1±11.6		73.0±12.1		<0.001
Systolic blood pressure – mmHg	123.5±17.2		121.2±15.7		129.0±20.0		121.8±17.6		<0.001
Medical history									
Hospitalization for heart failure	271 (36.8)		1687 (50.0)		78 (42.6)		161 (50.3)		<0.001
Atrial fibrillation	217 (29.5)		1345 (39.8)		56 (30.6)		138 (43.1)		<0.001
Diabetes	243 (33.0)		1447 (42.8)		70 (38.3)		168 (52.5)		<0.001
	**Placebo**	**Dapagliflozin**	**Placebo**	**Dapagliflozin**	**Placebo**	**Dapagliflozin**	**Placebo**	**Dapagliflozin**	**Int. P value**
**Efficacy**									
**Primary composite outcome**									
Number	45	28	392	297	11	8	35	46	
Rate	9.0 (6.7-12.0)	5.2 (3.6-7.5)	17.1 (15.5-18.9)	12.7 (11.3-14.2)	8.3 (4.6-14.9)	5.6 (2.8-11.2)	16.0 (11.5-22.2)	21.7 (16.3-29.0)	
Unadjusted HR	0.57 (0.36-0.92)		0.74 (0.64-0.86)		0.70 (0.28-1.78)		1.38 (0.88-2.15)		0.056
Adjusted HR	0.53 (0.32-0.86)		0.73 (0.63-0.85)		0.27 (0.08-0.88)		1.49 (0.92-2.40)		
**Hospitalization or urgent HF visit**									
Number	28	15	252	186	3	1	28	31	
Rate	5.6 (3.9-8.1)	2.8 (1.7-4.6)	11.0 (9.7-12.4)	7.9 (6.9-9.1)	2.3 (0.7-7.0)	0.7 (0.1-4.9)	12.8 (8.8-18.5)	14.6 (10.3-20.8)	

Unadjusted HR	0.49 (0.26-0.91)		0.72 (0.60-0.87)		0.43 (0.04-4.18)		1.16 (0.69-1.95)		0.20
Adjusted HR	0.43 (0.22-0.84)		0.71 (0.59-0.86)		-		1.28 (0.73-2.24)		
**CV death**									
Number	25	16	208	169	9	7	23	30	
Rate	4.8 (3.2-7.1)	2.9 (1.8-4.7)	8.4 (7.3-9.6)	6.8 (5.9-8.0)	6.6 (3.4-12.7)	4.8 (2.3-10.1)	9.9 (6.6-14.8)	13.2 (9.2-18.8)	
Unadjusted HR	0.61 (0.32-1.14)		0.82 (0.67-1.00)		0.71 (0.26-1.95)		1.31 (0.76-2.26)		0.28
Adjusted HR	0.60 (0.31-1.14)		0.83 (0.68-1.02)		0.24 (0.06-0.97)		1.46 (0.81-2.63)		
**Hospitalization for HF or CV death**									
Number	44	27	388	294	11	8	34	46	
Rate	8.7 (6.5-11.7)	5.0 (3.4-7.3)	16.9 (15.3-18.6)	12.5 (11.1-14.0)	8.3 (4.6-14.9)	5.6 (2.8-11.2)	15.5 (11.0-21.6)	21.7 (16.3-29.0)	
Unadjusted HR	0.57 (0.35-0.92)		0.74 (0.64-0.86)		0.70 (0.28-1.78)		1.42 (0.91-2.23)		0.041
Adjusted HR	0.52 (0.32-0.87)		0.73 (0.63-0.86)		0.27 (0.08-0.88)		1.56 (0.97-2.52)		
**Worsening renal function**									
Number	4	2	28	18	0	0	5	6	
Rate	0.8 (0.3-2.2)	0.4 (0.1-1.5)	1.2 (0.8-1.7)	0.8 (0.5-1.2)	-	-	2.2 (0.9-5.3)	2.8 (1.3-6.2)	
Unadjusted HR	0.49 (0.09-2.69)		0.64 (0.35-1.15)		-		1.35 (0.41-4.49)		0.69
Adjusted HR	0.11 (0.00-2.49)		0.66 (0.36-1.19)		-		1.51 (0.42-5.46)		
**Death from any cause**									
Number	31	23	249	204	10	9	30	32	
Rate	5.9 (4.2-8.4)	4.1 (2.8-6.2)	10.1 (8.9-11.4)	8.3 (7.2-9.5)	7.3 (4.0-13.7)	6.2 (3.2-11.9)	12.9 (9.0-18.4)	14.0 (9.9-19.9)	
Unadjusted HR	0.71 (0.41-1.21)		0.82 (0.68-0.99)		0.89 (0.36-2.19)		1.07 (0.65-1.76)		0.70
Adjusted HR	0.72 (0.42-1.26)		0.84 (0.70-1.01)		0.41 (0.14-1.26)		1.16 (0.68-1.98)		
**Safety**									
Discontinuation due to adverse event – no. (%)	19 (5.2)	12 (3.3)	82 (4.8)	83 (4.9)	4 (4.5)	1 (1.1)	6 (3.8)	10 (6.2)	0.19
Volume depletion – no. (%)	31 (8.5)	16 (4.3)	103 (6.1)	134 (8.0)	5 (5.6)	3 (3.2)	14 (8.8)	16 (9.9)	0.019
Renal adverse event – no. (%)	21 (5.8)	8 (2.2)	125 (7.4)	116 (6.9)	5 (5.6)	1 (1.1)	15 (9.4)	21 (13.0)	0.023

Data are presented as mean ± standard deviation or median (interquartile range) for continuous measures and number (%) for categorical variables.

Rates are per 100-patient years.

All unadjusted models include prior heart failure hospitalization (except for death from any cause and worsening renal function) and trial stratification.

Unadjusted model for worsening renal function includes estimated glomerular filtration rate.

All adjusted models include age, sex, race, New York Heart Association functional class, left ventricular ejection fraction, heart failure etiology, NT-proBNP (log), heart rate, systolic blood pressure, estimated glomerular filtration rate, atrial fibrillation and (except for death from any cause) prior heart failure hospitalization.

Supplemental Table II. Furosemide doses and changes in furosemide dose from baseline in CHARM (Added and Alternative)

	Placebo	Candesartan	All	P value	Odds ratio (95% CI)
**Baseline**					
Dose in all patients – mg	40.0 (0.0-80.0)	40.0 (0.0-80.0)	40.0 (0.0-80.0)	0.78	-
(n=4576)	51.0±60.5	53.5±73.0	52.2±67.0	0.22	
Dose in patients on furosemide – mg	40.0 (40.0-80.0)	40.0 (40.0-80.0)	40.0 (40.0-80.0)	0.53	-
(n=3220)	72.4±60.4	76.1±76.6	74.2±69.0	0.14	
**6 months**					
Dose in all patients – mg	40.0 (0.0-80.0)	40.0 (0.0-80.0)	40.0 (0.0-80.0)	0.32	-
(n=4332)	52.4±63.8	51.5±69.7	51.9±66.9	0.65	
Dose in patients on furosemide – mg	40.0 (40.0-80.0)	40.0 (40.0-80.0)	40.0 (40.0-80.0)	0.51	-
(n=3012)	74.8±64.3	74.6±73.0	74.7±68.8	0.93	
Decrease	170 (8.0)	253 (11.5)	423 (9.8)	<0.001	1.46 (1.18-1.81)
Increase	263 (12.3)	215 (9.8)	478 (11.0)	0.008	0.77 (0.63-0.93)
No change	1701 (79.7)	1730 (78.7)	3431 (79.2)	0.42	0.96 (0.83-1.12)
**14 months**					
Dose in all patients – mg	40.0 (0.0-80.0)	40.0 (0.0-80.0)	40.0 (0.0-80.0)	0.13	-
(n=4069)	51.9±64.8	48.7±66.2	50.2±65.5	0.12	
Dose in patients on furosemide – mg	40.0 (20.0-80.0)	40.0 (25.0-80.0)	40.0 (40.0-80.0)	0.017	-
(n=2778)	75.8±65.7	71.5±69.3	73.6±67.6	0.092	

Decrease	240 (12.0)	333 (16.1)	573 (14.1)	<0.001	1.39 (1.15-1.68)
Increase	344 (17.2)	277 (13.4)	621 (15.3)	<0.001	0.74 (0.62-0.88)
No change	1412 (70.7)	1463 (70.6)	2875 (70.7)	0.91	1.01 (0.88-1.16)
**18 months**					
Dose in all patients – mg	40.0 (0.0-80.0)	40.0 (0.0-80.0)	40.0 (0.0-80.0)	0.06	-
(n=3922)	51.0±63.3	48.4±67.5	49.7±65.5	0.2	
Dose in patients on furosemide – mg	40.0 (40.0-80.0)	40.0 (40.0-80.0)	40.0 (40.0-80.0)	0.017	-
(n=2658)	74.8±64.0	71.9±71.2	73.3±67.8	0.27	
Decrease	254 (13.2)	357 (17.9)	611 (15.6)	<0.001	1.44 (1.20-1.74)
Increase	366 (19.0)	290 (14.6)	656 (16.7)	<0.001	0.73 (0.61-0.86)
No change	1309 (67.9)	1346 (67.5)	2655 (67.7)	0.83	1.00 (0.87-1.15)

Data are presented as mean ± standard deviation or median (interquartile range) for continuous measures and number (%) for categorical variables.

Supplemental Figure I. Changes in loop diuretic dose according to diuretic therapy at baseline

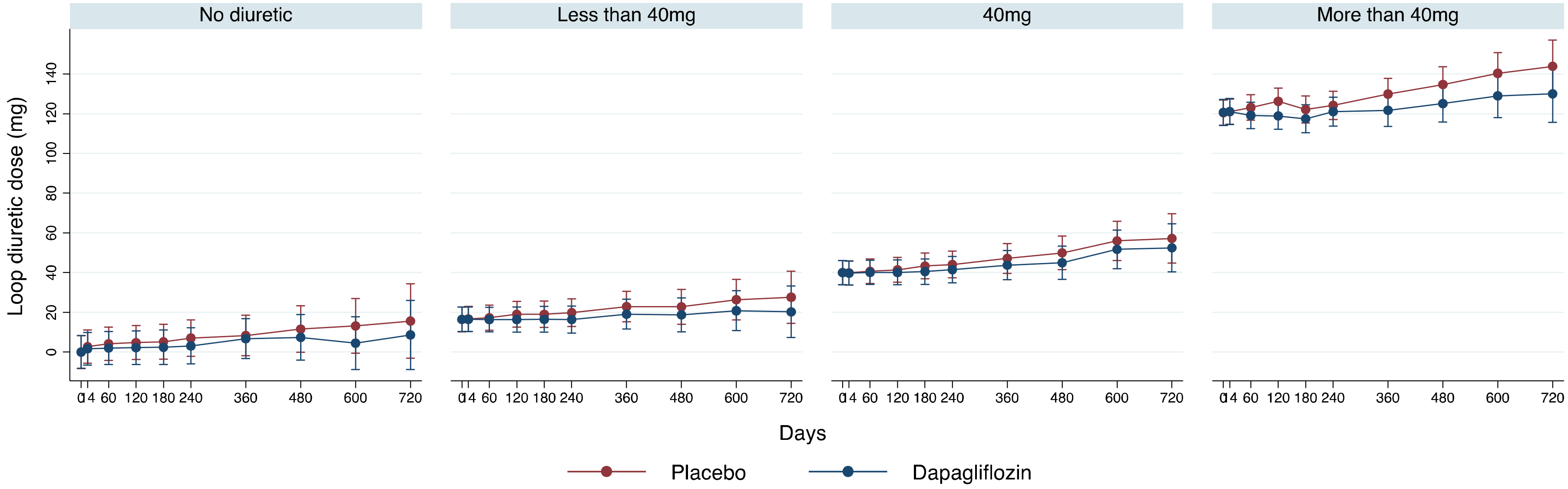

Values shown are estimates ± standard errors.

Supplemental Figure II. Change in furosemide dose over time in CHARM (Added and Alternative)

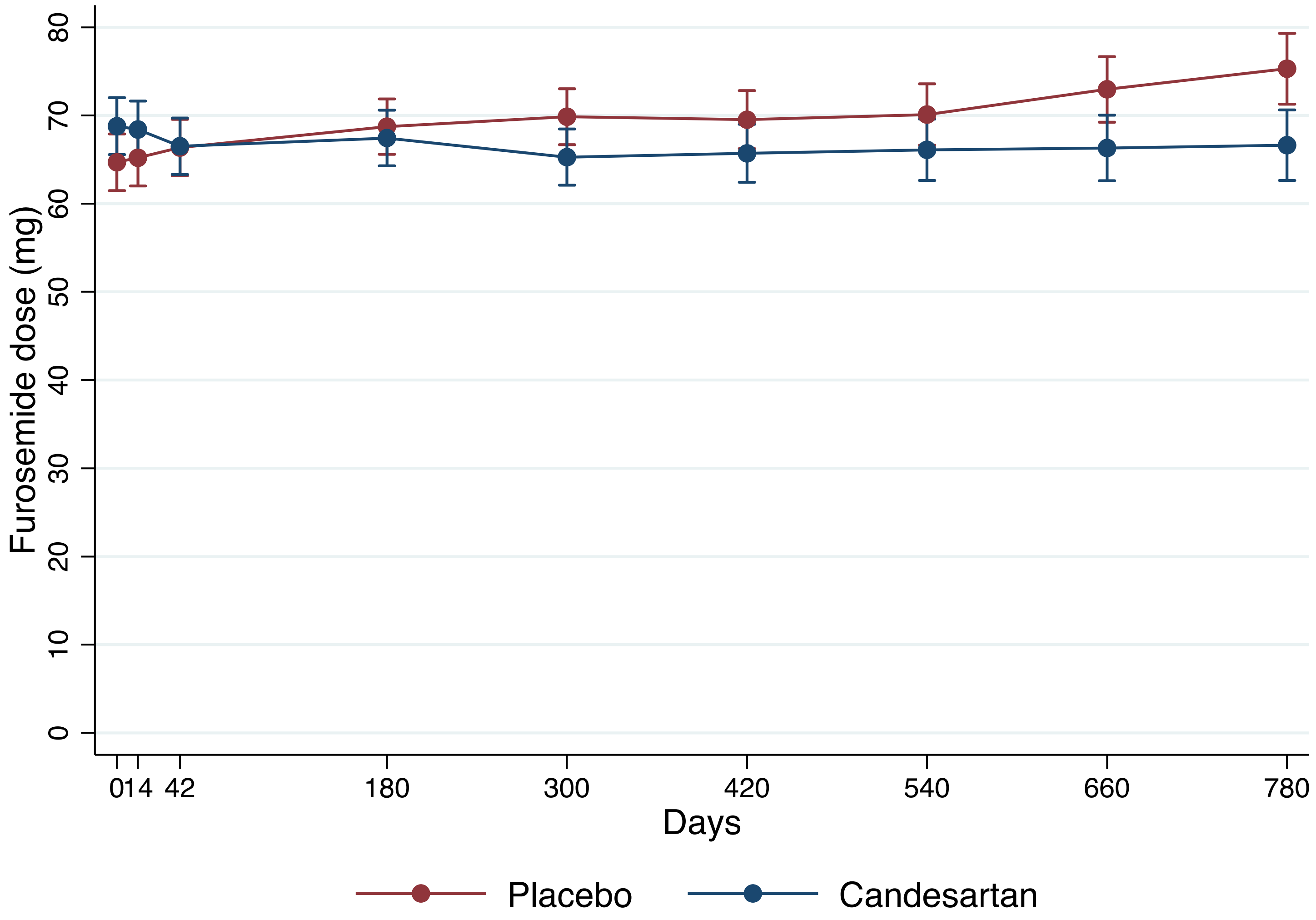

Values shown are estimates ± standard errors.

Supplemental Figure III. Changes in loop diuretic dose from 6 to 12 months (subsequent change) according to change in loop diuretic dose from baseline to 6 months (initial change)

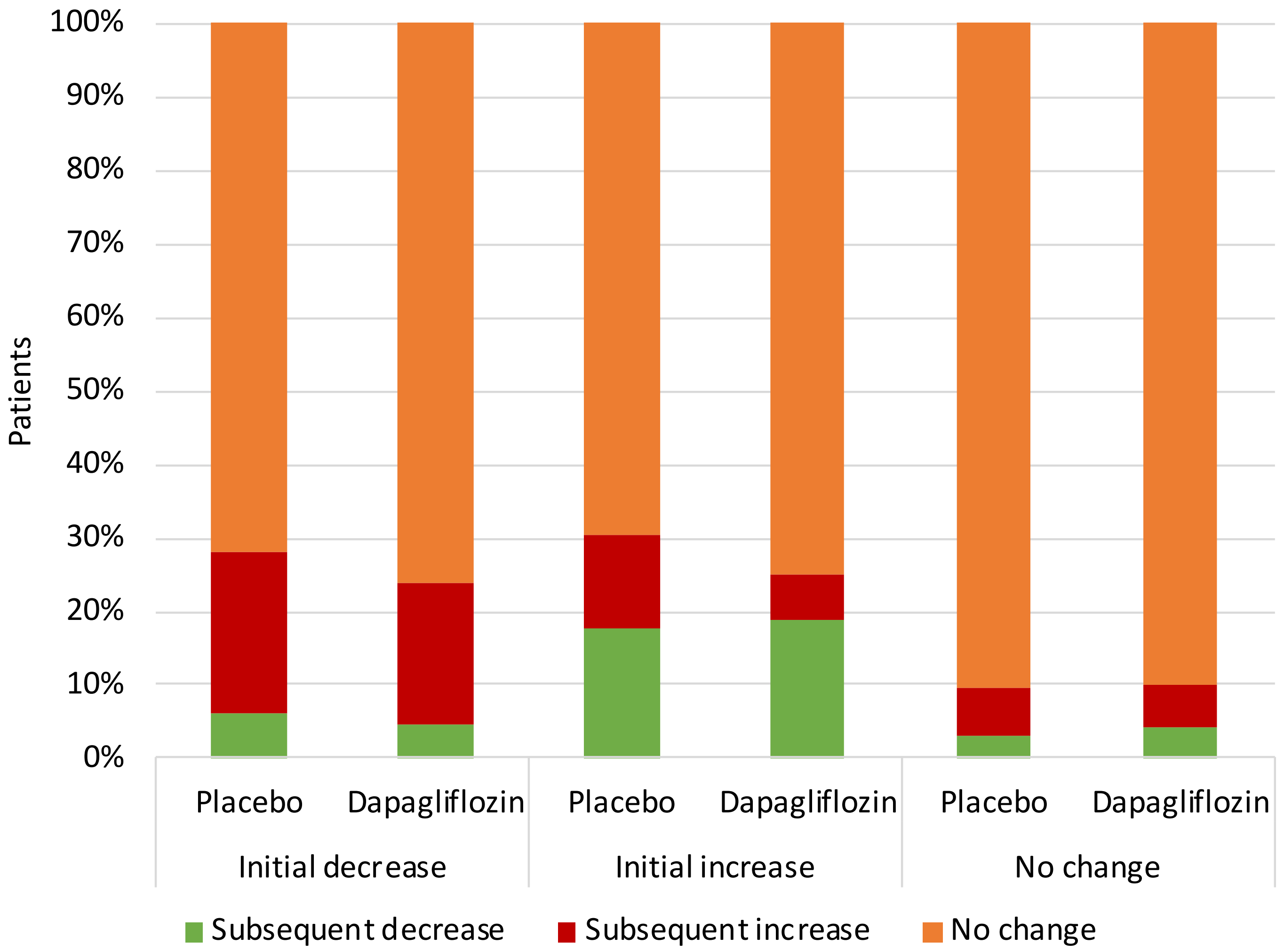

Supplemental Figure IV. Outcomes according to diuretic therapy at baseline

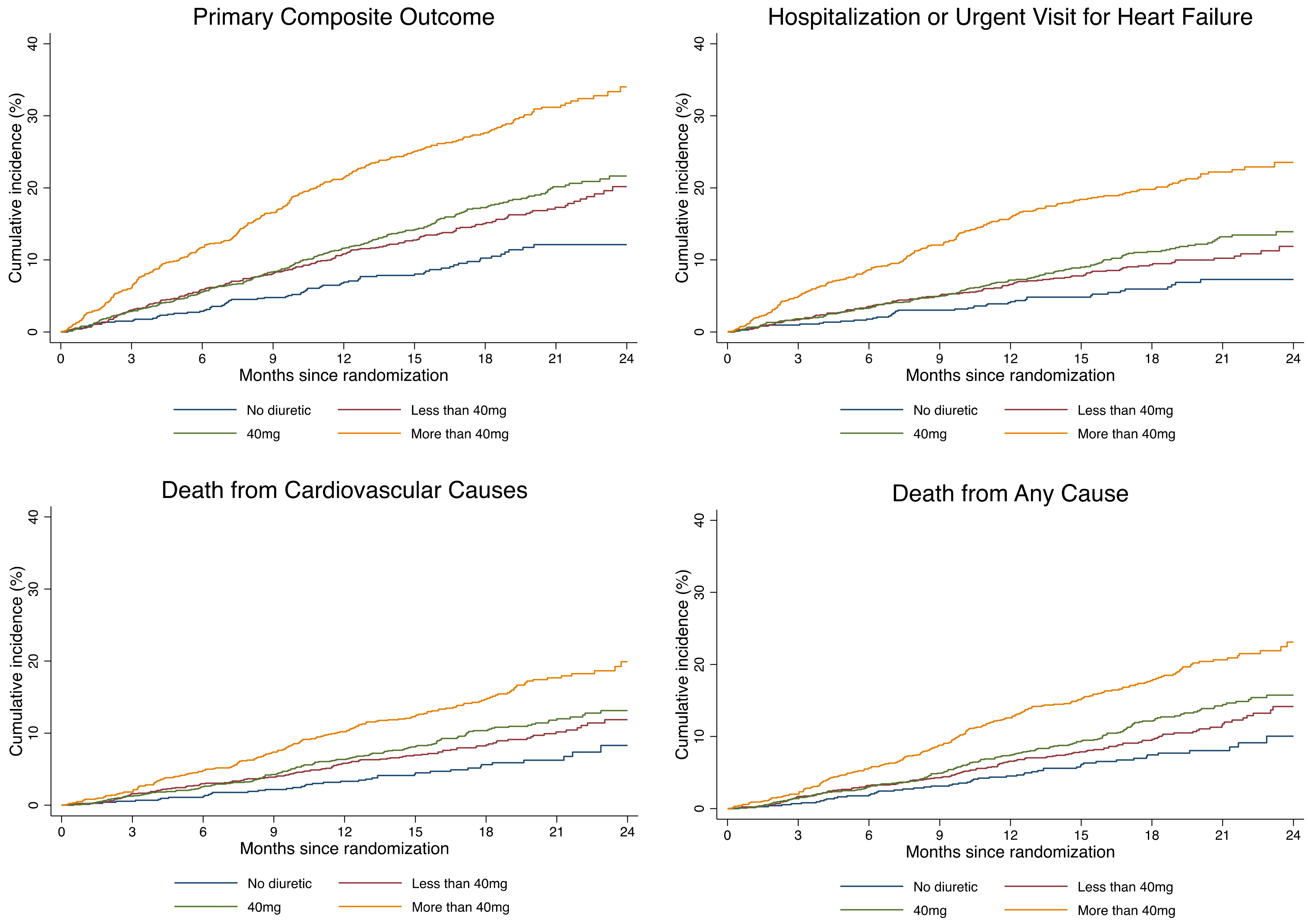

Supplemental Figure V. Outcome according to change in loop diuretic dose from baseline to 6 months

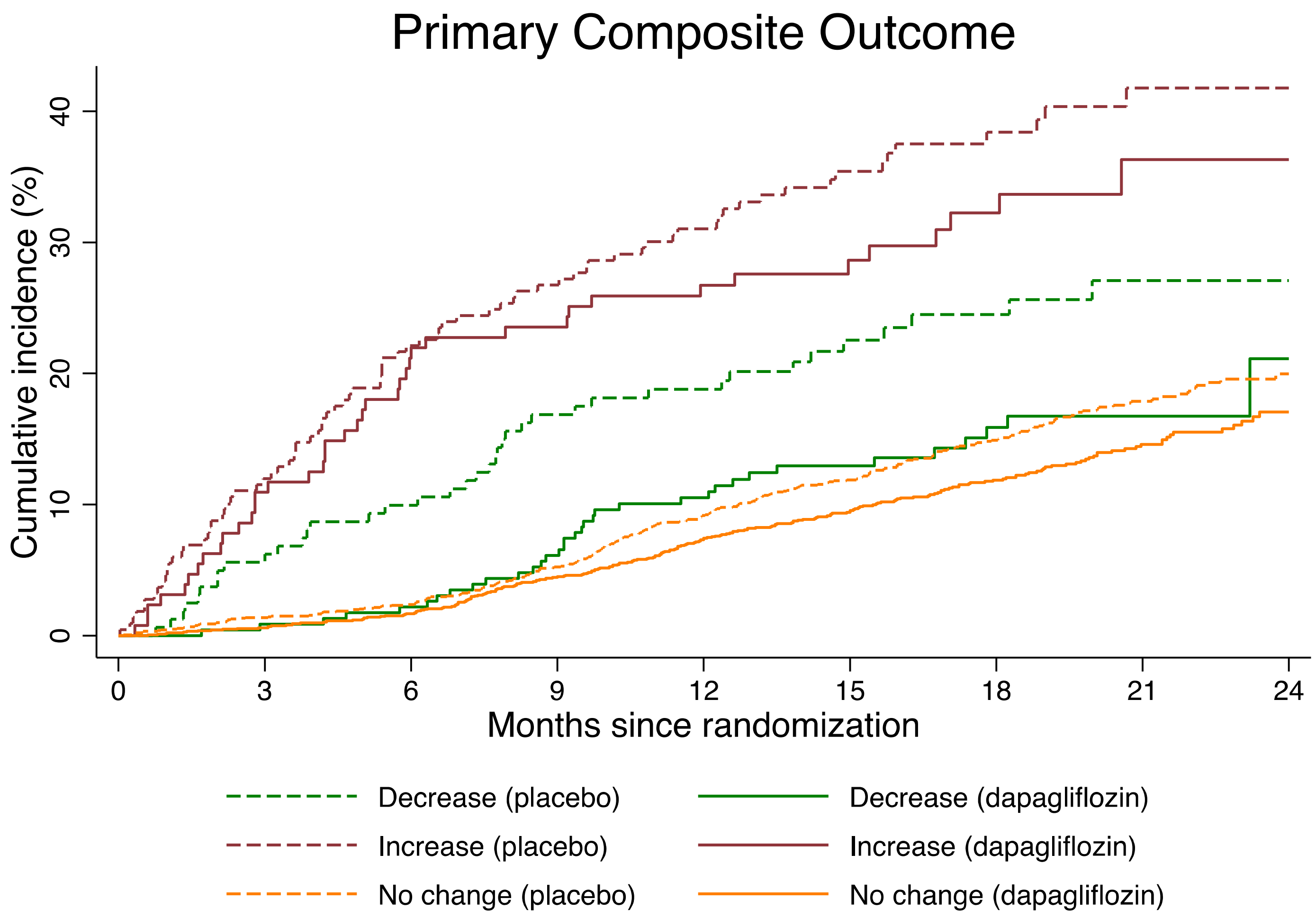

Supplemental Figure VI. Change in hematocrit according to change in loop diuretic dose from A) baseline to 6 months and B) baseline to 12 months

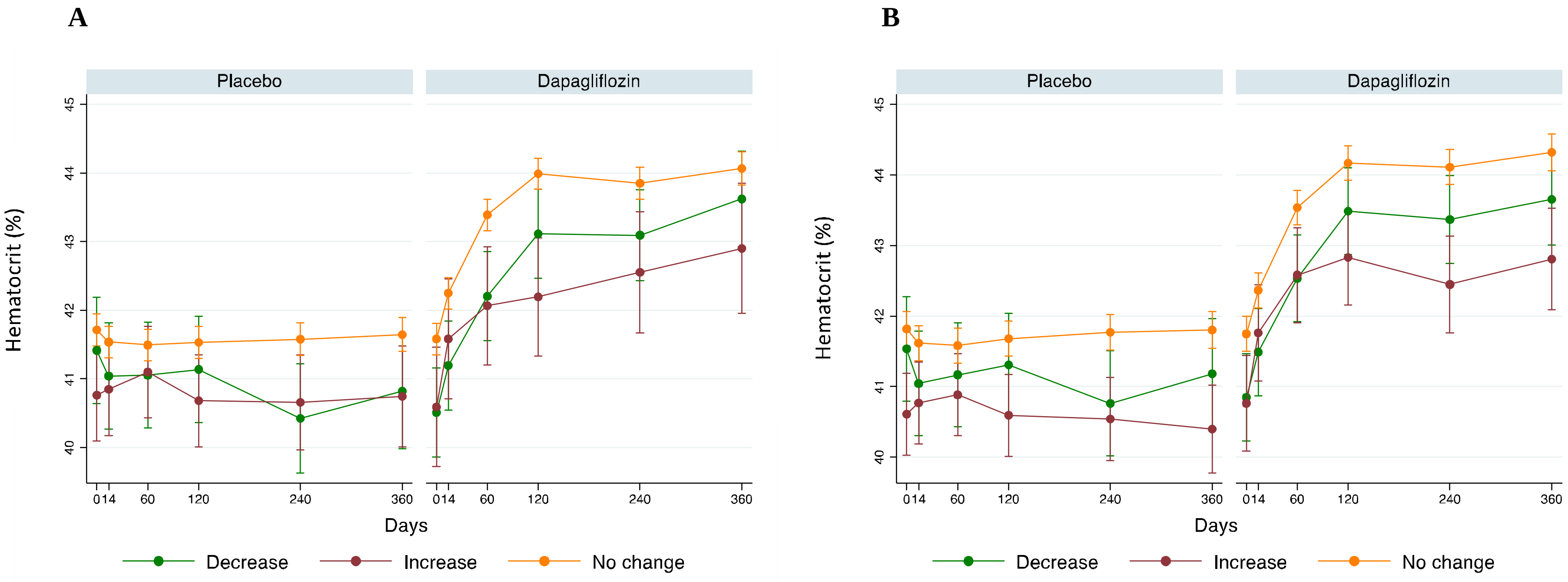

Values shown are estimates ± standard errors.
